# 2460: Change in duration of postoperative antibiotic prophylaxis in esophagectomy patients: Outcomes in a single academic institution

**DOI:** 10.1017/cts.2017.280

**Published:** 2018-05-10

**Authors:** Sue Wang, Gavitt A. Woodard, Calixto-Hope Lucas, Stanley J. Rogers, David M. Jablons

## Abstract

OBJECTIVES/SPECIFIC AIMS: Ivor-Lewis esophagectomy (ILE) is an invasive surgical procedure with a high incidence of postoperative pneumonia. Antibiotic prophylaxis could reduce respiratory infections but increase *Clostridium difficile* and antibiotic resistance. Our institution reduced the duration of piperacillin-tazobactam prophylaxis following ILE from 4 to 1 day or less in January 2015. We evaluated short-term outcomes in ILE patients before and after this institutional change. METHODS/STUDY POPULATION: Retrospective cohort study of all ILE patients from 2012 to 2016. We confirmed antibiotic duration directly from nursing medication administration records. The primary outcomes of this study were rates of *C. difficile* and postoperative pneumonia. Secondary outcomes include other infection, length of hospital stay, and readmission within 30 days. We used logistic regression to analyze impact of days of antibiotics and χ^2^ or Fischer exact tests for categorical variables. RESULTS/ANTICIPATED RESULTS: Of 104 ILE patients, 40.4% (n=42) were after January 2015, 11.5% developed pneumonia and 5.8% developed *C. difficile* colitis. ILE patients received more days of antibiotics before the institutional change compared with after (6.1 vs. 2.9 d, *p*<0.01). For a 1-day increase in antibiotic duration, the odds of acquiring *C. difficile* increased significantly by 1.2 (*p*=0.03). Before compared with after the institutional change, rates of *C. difficile* were 8.1% Versus 2.4% (*p*>0.2), rates of pneumonia were 11.3% Versus 11.9% (*p*>0.2), and length of stay was 10.9 Versus 10.5 days (*p*>0.2), respectively. DISCUSSION/SIGNIFICANCE OF IMPACT: Institutional policy can have an impact on patient outcomes. Antibiotic stewardship is associated with reduced rates of inpatient *C. difficile*. Our study suggests reduced antibiotics are not associated with pneumonia, although larger studies are necessary to confirm this finding. Surgeons should consider the benefit of decreased rates of *C. difficile* before administering prolonged antibiotic prophylaxis following esophagectomies.

